# Living on fire: Deactivating fire coral polyps for larval settlement and symbiosis in the fire coral‐associated barnacle *Wanella milleporae* (Thoracicalcarea: Wanellinae)

**DOI:** 10.1002/ece3.9057

**Published:** 2022-07-05

**Authors:** Fook‐Choy Yap, Jens T. Høeg, Benny K. K. Chan

**Affiliations:** ^1^ Biodiversity Research Center Academia Sinica Nangang Taiwan; ^2^ Department of Biology, Marine Biological Section University of Copenhagen Copenhagen Denmark; ^3^ Present address: Department of Biological Science, Faculty of Science Universiti Tunku Abdul Rahman, Jalan Universiti, Bandar Barat Perak Malaysia

**Keywords:** exploratory phase, fire coral, settlement, stinging polyps, symbiosis

## Abstract

Symbiosis is increasingly recognized as being an important component in marine systems, and many such relationships are initiated when free‐swimming larvae of one partner settle and become sedentary on a host partner. Therefore, several crucial questions emerge such as the larva’s mechanism of locating a host, selection of substratum and finally settlement on the surface of its future partner. Here, we investigated these mechanisms by studying how larvae of the fire coral‐associated barnacle *Wanella milleporae* move, settle and establish symbiosis with their host, *Millepora tenera*. Cyprids of *W. milleporae* possess a pair of specialized antennules with bell‐shaped attachment discs that enable them to explore and settle superficially on the hostile surface of the fire coral. Intriguingly, the stinging polyps of the fire coral remain in their respective pores when the cyprids explore the fire coral surface. Even when cyprids come into contact with the nematocysts on the extended stinging polyps during the exploratory phase, no immobilization effects against the cyprids were observed. The exploratory phase of *Wanella* cyprids can be divided into a sequence of wide searching (large step length and high walking speed), close searching (small step length and low speed) and inspection behavior, eventually resulting in permanent settlement and metamorphosis. After settlement, xenogeneic interactions occur between the fire coral and the newly metamorphosed juvenile barnacle. This involved tissue necrosis and regeneration in the fire coral host, leading to a callus ring structure around the juvenile barnacle, enhancing survival rate after settlement. The complex exploratory and settlement patterns and interactions documented here represent a breakthrough in coral reef symbiosis studies to show how invertebrates start symbiosis with fire corals.

## Graphical abstract

Most of previous studies on coral reef symbiotic fauna were focused on diversity and host usages. How these invertebrates start their life with their host is almost unknown. Our study provides a breakthrough in the field of coral reef symbiosis by examining how fire coral associated barnacles explore, settle, metamorphose to start the symbiotic life with fire corals.

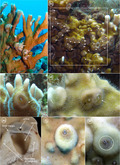

## 1 INTRODUCTION

Coral reefs have been regarded as a complex ecosystem that serves as a habitat for one‐third of the known marine species (Brandl et al., 2019; Knowlton, 2001; Knowlton et al., 2010; Reaka‐Kudla, 1997; Wagner et al., 2020). Within coral reefs, scleractinian and hydrocorals contribute to reef accretion and the dynamics of community assemblages (Dubé et al., 2019; Lewis, 2006). In addition, most marine invertebrates found on reefs live in symbiosis with coral or hydrocoral hosts, contributing significantly to the high biodiversity (Stella et al., 2011). We define symbiosis

as “any close association between dissimilar organisms living together irrespective of how harmful or beneficial it may be for either” (Dreyer & Chan, 2020). Before forming symbiotic relationships, these invertebrate larvae must explore, locate and settle on their specific hosts. However, the exploratory behavior and settlement patterns in these symbiotic species remain elusive to date. The lack of studies in this regard is probably due to the difficulty in culturing corals and their associated invertebrate symbionts under laboratory conditions.

**FIGURE 1:**
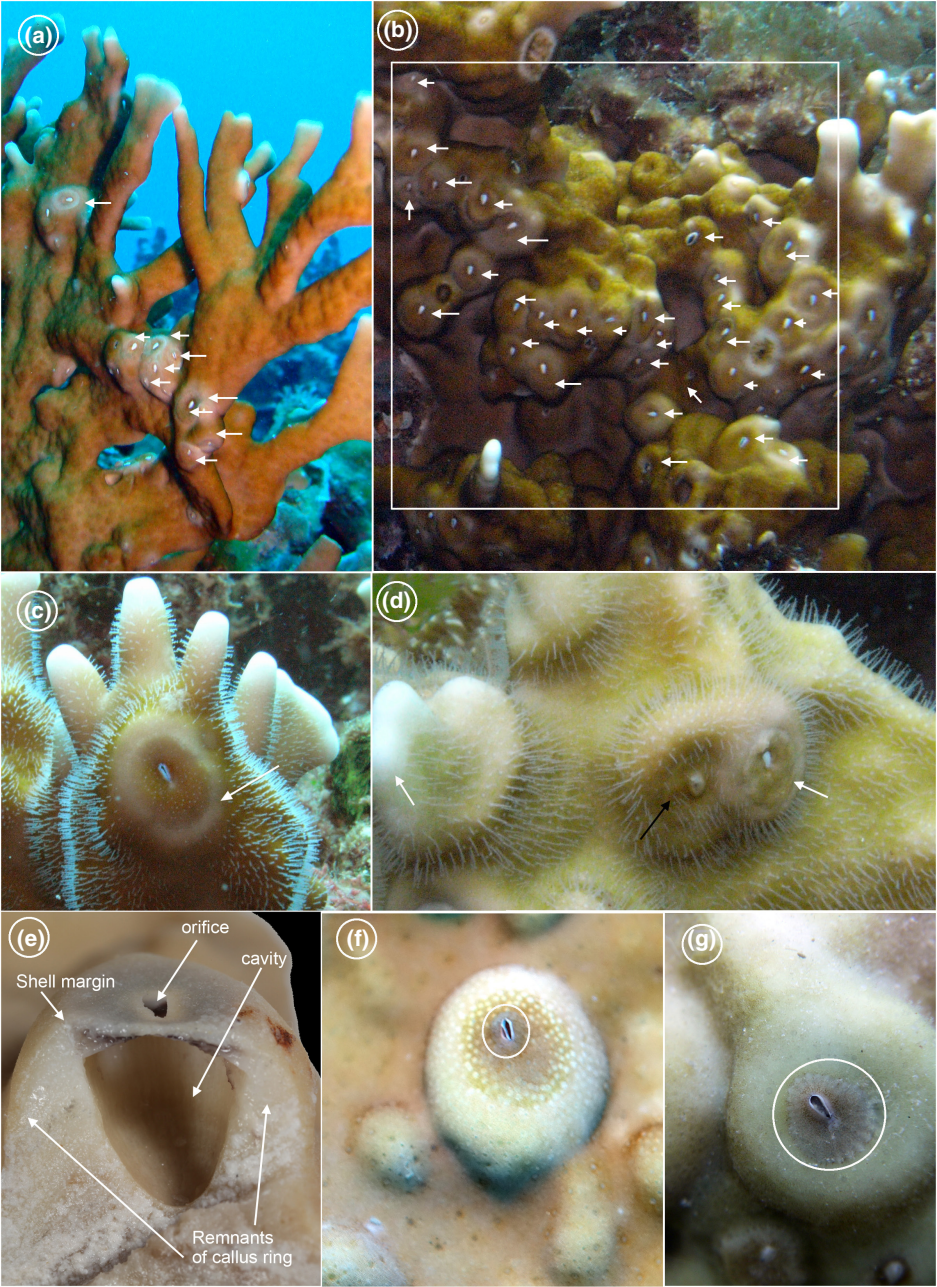
(a) Fire corals and their associated barnacles (indicated by white arrows). (b) A high‐density colony of *Wanella milleporae* (indicated by white arrows) in a 5 × 5 cm quadrat area. (c) Magnified view of *Wanella milleporae*. Note that the fire corals have stinging polyps, which can extend armed with venom containing nematocysts to attack a variety of organisms. There is often a “callus” around each barnacle, where the density of stinging polyps is less than that of the surroundings. (d) One live (indicated by white arrow) and one dead (indicated by black arrow) *Wanella milleporae* on fire corals. Note the dead individual already has fire coral overgrowth on its shells enclosing the operculum. Stinging polyps can be seen on the shell surface of the overgrown coral and also on the remnants of callus on the live individual. (e) Longitudinal section of *Wanella* on the remnants of the callus ring (barnacle somatic body removed), showing the shell cavity embedded in the remnants of the callus ring. (f) An early settled *Wanella milleporae* (highlighted by white circle) observed in the field. Note the callus is formed underneath the barnacle, which appears to be pale in color and free of zooxanthellae. (g) A juvenile of *Wanella milleporae*, with its shells expanding horizontally, without coral overgrowth, remnants of the callus ring already recolonized by zooxanthellae

Hydrocorals are non‐scleractinian corals belonging to the class Hydrozoa. Species of *Millepora* are well known for their defense mechanisms, having distinct pores on the coenosteal surface of the calcareous skeleton to accommodate the feeding (gastrozooids) and defense (dactylozooids) polyps (Dubé et al., 2019; Lewis, 2006). Gastrozooids are cylindrical with a central mouth and dactylozooids are long and slender but without mouths. The defense polyps of *Millepora* have clusters of capitate tentacles which possess encapsulated nematocysts filled with highly toxic venom (García‐Arredondo et al., 2012; Hernández‐Matehuala et al., 2015; Moats, 1992) and can also be used for food capture (de Kruijf, 1975; Lewis, 2006). The polyp extensions of *Millepora* can be triggered by the presence of prey or electrical signals (de Kruijf, 1976; Lewis, 2006). The polyps can expand during both days and nights (Lewis, 1989). The various *Millepora* species have gained the name “fire coral” because envenomation by their nematocysts can cause serious localized symptoms by skin irritation, burning or stinging pain, erythema, edema and urticaria in human skin (Kropp et al., 2018; Ozbek et al., 2009). Lethal or harmful to a variety of vertebrate and invertebrate organisms (Lewis, 2006), the toxic defense mechanism of the fire coral creates an unappealing habitat for reef organisms’ sheltering. Nevertheless, some marine invertebrates, such as barnacles *Wanella milleporae*, Serpulid polychaetes *Spirobranchus polycerus*, amphipods *Stenothoe valida* and snapping shrimps *Alpheus obesomanus*, are still found living as their symbionts (Barnard, 1970; Kopp, 1987; Lewis, 1989; Lewis, 1998a,b; Marsden, 1992). Among these associated invertebrates, the barnacle *Wanella milleporae* is the most abundantly associated invertebrate that lives on fire corals of the Indo‐Pacific. The barnacle body embeds with a cavity within the fire coral skeleton (Lewis, 2006; Tsang et al., 2009), and thus, suggests an intimate symbiotic interaction with fire corals (Figure 1a). The opercular plate lies flat with the fire coral surface (Chan et al., 2013). The symbiotic relationship between *Wanella* and *Millepora* is still controversial. Some studies suggest that the relationship is mutualistic. *Millepora* provides refuge for *Wanella* against predation risks. Nitrogen and phosphorus excreted by the barnacles can be absorbed by the fire coral zooxanthellae to enhance photosynthetic activity (Achituv et al., 1997; Achituv & Mizrahi, 1996; Cook et al., 1991). However, Vago et al. (1994) suggested that high‐density colonization of *Wanella* on fire corals can cause skeleton deformation, but whether there are damaging effects on fire corals is still unknown. In the field, fire corals with a high density of colonizing barnacles still possess a strong skeleton (Figure 1). In the present study, we consider the symbiotic relationships between *Wanella* and fire corals are mutualistic based on the evidence provided in Cook et al. (1991) and Achituv and Mizrahi (1996).

To start a symbiotic life, larvae of *W. milleporae* must first locate the fire corals, then possibly need to evade, tolerate or neutralize the defense polyps; furthermore, they explore the fire coral surface and finally settle and adapt to the environment of fire corals. In barnacles, the terminal larval stage, cyprid, is non‐feeding and structurally highly adapted to the task of locating a suitable substratum while swimming, exploring its surface and finally attaching permanently. The cyprid is equipped with a pair of highly modified antennules that enable them to walk bipedally over a substratum while using an array of sensory organs to choose the final attachment site and initiate metamorphosis (Aldred et al., 2013; Chan et al., 2014; Crisp, 1976; Lagersson & Høeg, 2002; Phang et al., 2008). The exploratory walking events on the surface are critical, specifically to symbiotic barnacles, allowing the cyprids to access the settlement surface conditions before permanent and irreversible attachment by cement secretion. Thus, the exploratory events of *W. milleporae* can lead to the initiation of symbiotic relationships.

Here, we are the first to successfully culture larvae of the fire coral‐associated barnacle *W. milleporae* and describe the movement, exploration, settlement and metamorphosis of cyprids on the fire coral host under laboratory conditions that lead to symbiotic life in coral reefs.

## 2 METHODS

### 2.1 Fire corals and associated barnacle sampling

The fire coral, *Millepora tenera* (previously known as *Millepora tenella*), and its associated barnacle, *Wanella milleporae,* were collected from Green Island, Taiwan via scuba diving at a depth of 10–20 m. The frequency of occurrence of *Wanella* on fire corals in Green Island and adjacent waters reached 100% in our surveys of 30 fire coral colonies (barnacle percentage cover on fire coral colonies ranged from 8% to 32%, the density of barnacles per colony can reach 31 individuals 25 cm^−2^; Figure 1b). Fire corals bearing their associated barnacles were collected using a hammer and a chisel. Additionally, the fire corals were photographed in situ for identification purposes. Two different sizes of fire coral fragments were collected: (1) Large fragments of fire corals (about 12–15 cm^2^) with their associated barnacles (about 7–9 individuals) for barnacle larval culturing; (2) small fragments of fire corals (about 5–7 cm^2^) with their associated barnacles (about 0–3 individuals) for settlement and metamorphosis experiments.

### 2.2 Larval rearing and culture

The collected fire corals and associated barnacles were transferred separately into a 7 L polycarbonate plastic container filled with filtered seawater. All cultures were maintained at 26°C in a 12:12 h light–dark cycle. The seawater was changed daily. Frozen planktons (collected using plankton net in shallow water) were supplied as food for the fire coral and associated barnacles after changing the seawater.

Newly released stage I nauplii of *W. milleporae* were collected within 2 weeks after collection with the aid of a pointed light source, since the larvae have positive phototaxis. The collected nauplii were transferred into sterile Petri dishes (5.5 and 9 cm in diameter) containing filtered seawater (filtration: 0.45 μm, salinity: 33%). Antibiotics (Penicillin and Streptomycin) were added into the larval culture to inhibit bacterial growth. The seawater was changed every 2 days to avoid accumulation of waste. The nauplii were fed with mixtures of microalgae (*Isochrysis* sp., *Chaetoceros* sp., *Skeletonema costatum*) after changing the seawater. The larval cultures in Petri dishes were maintained at 26°C in a 12:12 h light–dark cycle. The cultures were continued until the larvae reached the cyprid stage.

### 2.3 Morphology of cyprid attachment organ

Scanning electron microscopy (SEM) was used to study the surface morphology of the cyprid’s antennular structures. Cyprids were relaxed in 8% magnesium chloride for 1 h, washed with 0.45 μm filtered seawater several times and then fixed with 2.5 glutaraldehyde (seawater base). The glutaraldehyde‐fixed cyprids were dehydrated through an ascending series of ethanol, critical point dried, mounted on aluminum stubs and sputter‐coated with gold. The gold‐coated cyprids were viewed using a FEI Quanta 200 scanning electron microscope operating at 20 kV.

### 2.4 Responses of fire corals towards barnacle nauplii and cyprids

To infer the defensive behaviors of the stinging polyps of fire corals towards the two larval forms of barnacles, we introduced nauplii and cyprids of *W. milleporae* to the fire corals. About 50 individuals of nauplii (stage II and VI) and 10 individuals of different age groups of cyprids (1‐, 2‐ and 3‐day‐old) were introduced to a small polycarbonate plastic container containing filtered seawater and a small piece of fire coral (about 5–7 cm^2^). The responses of the stinging polyps of fire corals towards the nauplii and cyprids of *W. milleporae* were recorded and photographed using a Leica M125 stereomicroscope (Leica, Germany) equipped with a custom‐made C‐mount and lens mount adapter attached to a Panasonic Lumix GH4 camera. An additional light‐emitting diode (LED) illuminator was used during the video recording. All photomicrographs and video recordings were processed using Adobe Photoshop CS6, Adobe Illustrator CS6 and Corel VideoStudio Ultimate 2020.

### 2.5 Exploratory walking, settlement and metamorphosis of barnacles on fire corals

We introduced cyprids to the fire coral host to describe the settlement and metamorphosis of *W. milleporae*. The cyprids (*n* = 10) were transferred into a small polycarbonate plastic container containing filtered seawater and a small fire coral piece. About half of the seawater in the settlement experiment was changed every 6–8 h. Permanent settlement of cyprids onto the fire coral occurred within 24 h. We discovered two types of reactions from the fire corals towards the newly metamorphosed juveniles. The mortality of juveniles caused by these reactions from the fire corals was recorded.

Settlement behaviors, cyprid metamorphosis and the responses of fire corals towards the juveniles were recorded and photographed using the methods above. These experiments were repeated three times for accuracy and precision. All settlement and metamorphosis data of cyprids were processed using the same software as mentioned above. The walking distances during the settlement behaviors (wide and close searching) were measured to determine the average distance for each antennular step. The data were then subjected to Welch unpaired t‐test to determine the differences between the walking event of the wide and close searching behaviors.

To determine the correlations between the larval age, walking behavior and settlement, we used different age groups (1‐ to 9‐day‐old) of cyprids (*n* = 10, replicate = 3). For each age group, the number of cyprids engaged in walking behavior and the number of cyprids settled on the fire corals were recorded to estimate the rate of walking and settlement of different age groups. The data were evaluated with a one‐way analysis of variance (ANOVA) and the pairwise comparisons of Tukey honestly significant difference (HSD) test at α‐level of 0.05. All statistical analyses were conducted in R (R Core Team, 2021) using the package ggplot2 (Wickham, 2016).

## 3 RESULTS

**FIGURE 2:**
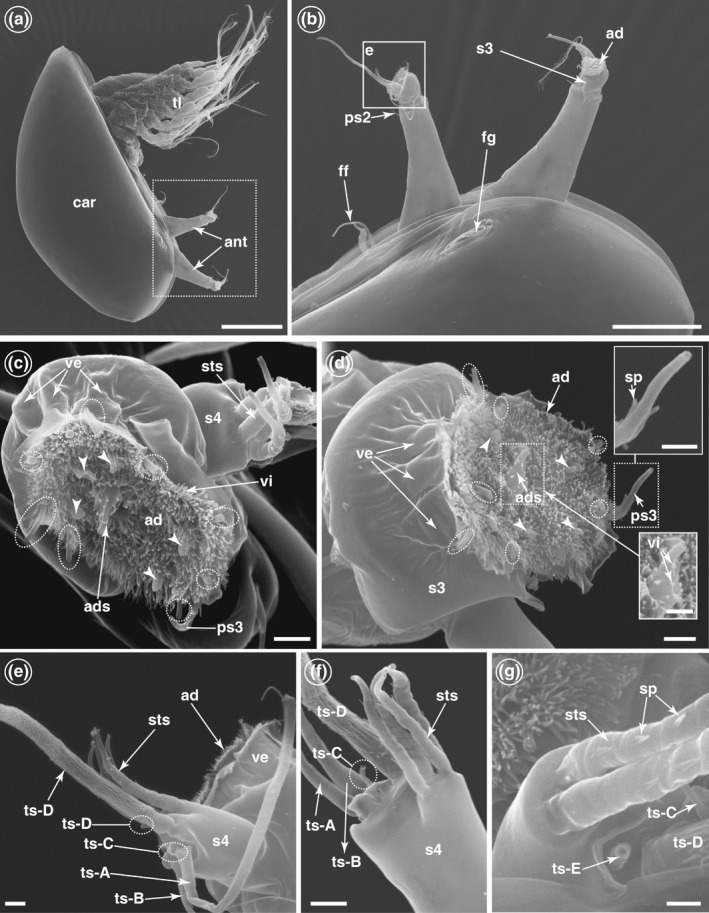
Scanning electron microscope (SEM) images of whole cyprid and its attachment organs of *Wanella milleporae*. (a) Cyprid possesses a pair of highly modified antennules (ant). (b) A bell‐shaped attachment disc (ad) located in the third antennular segment (s3). (c) The attachment disc covered with microcuticular villi (vi). The axial disc seta (ads) protrudes from the medial region of the attachment disc and is surrounded by five cuticular tubes (arrowhead). Fourth antennular segment (S4) extends from the lateral side of the third antennular segment. (d) The attachment disc encircled with cuticular structures known as velum (ve). A series of radial setae (dotted circle) found at the periphery of the attachment disc. Inset: The axial disc seta (ads) is also covered by short microcuticular villi. The third antennular segment bears a post‐axial seta (PS3) covered with lateral spines (sp). (e) The fourth antennular segment carries five terminal setae (ts‐a, ts‐b, ts‐c, ts‐d, ts‐e) and four subterminal (sts) setae. (f) Detailed view of the terminal and subterminal setae. (g) Enlarged view of subterminal setae. Car; cyprid carapace; ff, frontal filaments; fg, frontolateral gland pore; ps2, post‐axial seta 2; tl, thoracic limbs. Scale bars, 100 μm (a), 50 μm (b), 2 μm (c, d, e, f); 1 μm (g, insets in d)

All larval stages of *Wanella milleporae*, comprised of six nauplius stages followed by the terminal cyprid stage, were successfully cultured. Development from nauplius 1 to cyprid took about 11 days to complete at 27°C. The SEM photomicrographs showed that the general morphology of the cyprid resembles most barnacle species. The cyprid body is enclosed in a smooth carapace without any sculpturing. It carries a pair of antennules in the anterior region and six pairs of thoracic limbs in the posterior region (Figure 2a). Both types of appendages can be retracted completely into the mantle cavity. The average length of the cyprid (carapace length) is 426 μm (*n* = 10, range 392–450 μm). Our study focused on the antennules, their sensory setae and the attachment organ (third segment), which play a crucial role in the exploratory and settlement behaviors.

### 3.1 Morphological features of the cyprid attachment organ

The antennules in *W. milleporae* cyprids exhibit the typical segmentation of barnacles (Bielecki et al., 2009; Lagersson & Høeg, 2002). The elongated second segment carries a single seta (post‐axial seta 2 (ps2)) at the distal end (Figure 2b). The third segment (the attachment organ) is nearly bell‐shaped with a slight constriction at the distal end (Figure 2b, d). The attachment disc is densely covered by microcuticular villi and encircled by a series of thin‐walled cuticular flaps (Figure 1c, d). The third segment carries a post‐axial seta (post‐axial seta 3 (ps3)) ventrodistally (Figure 2d). An axial disc seta (ads) extends out from the medial region of the attachment disc via an opening pore (Figure 2c, d). The ADS is covered with a few short microcuticular villi (Figure 2c, d) and terminates in a pore. It is surrounded by five conspicuous cuticular tubes (Figure 2c, d, white arrowhead), originating from the cuticle of the attachment disc. These five cuticular tubes have wrinkled surfaces with large terminal pores, which probably serve as pores for the permanent adhesive secretions of the cyprid (Nott & Foster, 1969; Walker & Yule, 1984). A series of radial setae (RDS1–RDS9) of variable length extends out from the periphery of the attachment disc (Figure 2c, d, dotted circle).

The trapezoid‐shaped fourth segment projects from the lateral side of the third (Figure 2c, e) and carries a subterminal and a terminal group of setae (Figure 2e‐g). Among the five terminal setae (ts‐A‐E), ts‐A and ts‐B are morphologically identical, both long and furnished (Figure 2e, f), with laterally extending setules in their distal part. The ts‐C and ts‐E are short with terminal pores (Figure 2f, g), while ts‐D is long, with a terminal pore and ornamented with irregular surface structures (Figure 2e, f). The four subterminal setae (sts) are identical to each other, with projecting lateral spines and terminal pores (Figure 2e‐g).

### 3.2 Responses of the fire coral polyps towards *Wanella milleporae* larvae

**FIGURE 3:**
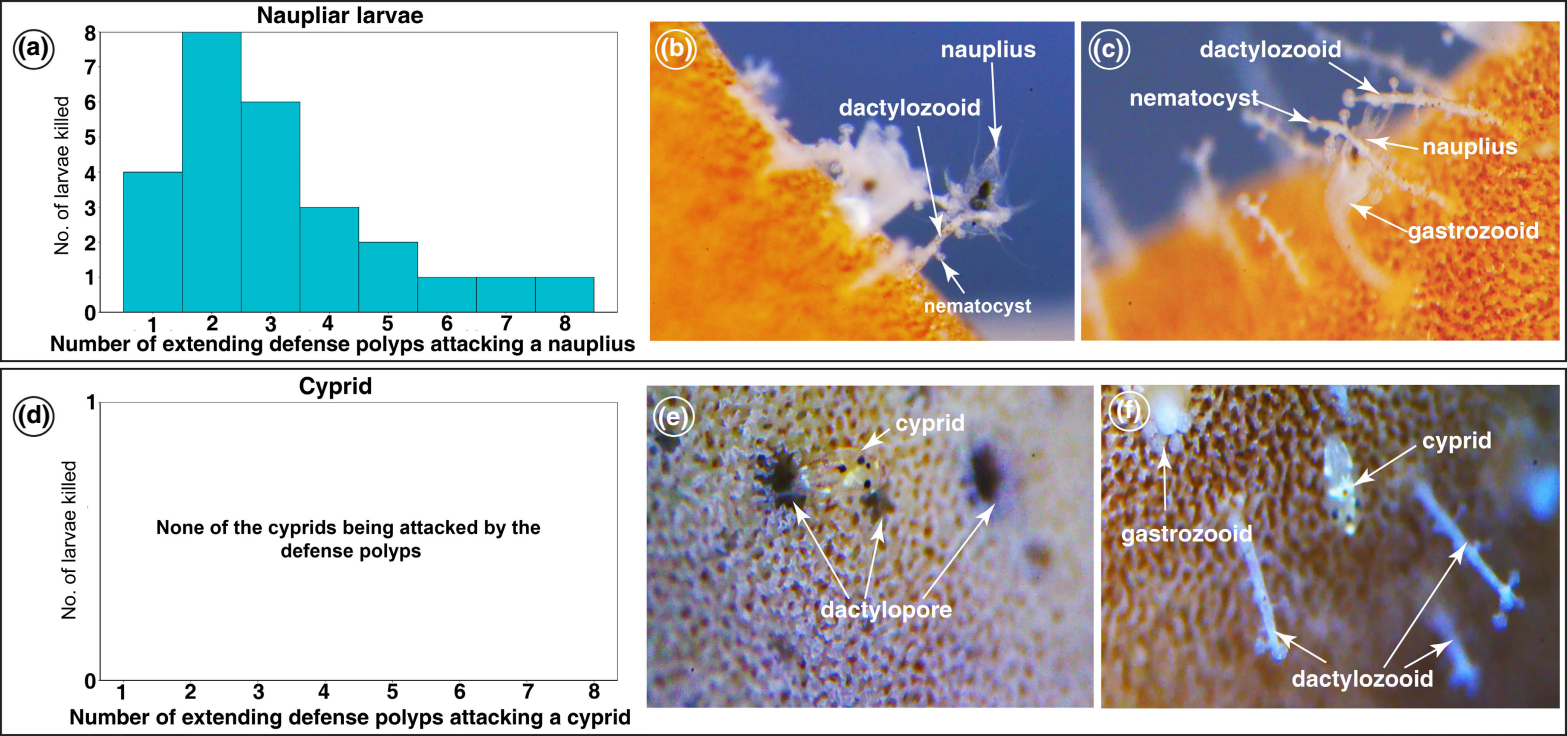
Responses of stinging polyps fire coral after exposure to the barnacle nauplii and cyprids of *Wanella milleporae*. (a) Frequencies of the number of dactylozooids (x axis) attacking naupliar larvae (Y‐axis – the number of larvae being attacked). When a nauplius swims close to the fire corals, they can be attacked by different numbers of stinging polyps. (b) The dactylozooids and gastrozooids extended from their respective pores after nauplii were exposed to the fire coral. The nauplius was incapacitated by the tentacles of nematocysts on the dactylozooids and was transferred to gastrozooids by dactylozooids. (c) The gastrozooid captured the incapacitated nauplius from the dactylozooid. (d) Frequencies of the number of dactylozooids attacking cyprids; the trials showed that no cyprids were attacked. (e) The dactylozooids and gastrozooids remained in their respective pores, and being inactive, during the cyprid exploratory behavior. (f) the dactylozooids are irresponsive towards the presence of *Wanella* barnacle cyprids, when cyprids walk through the gap between the dactylozooids

Exposure to nauplii and cyprids of *Wanella* stimulated different responses in the polyps of the fire corals. The stinging polyps of fire corals were highly responsive towards the barnacle nauplii (Figure 3a‐c). We observed that both dactylozooids and gastrozooids slowly extended from their respective pores after exposure to the nauplii (Figure 3b, c). The dactylozooids actively swayed towards the nauplii, while the gastrozooids were still extending (Figure 3b, c; Video S1). Once a nauplius was immobilized, the gastrozooid responded by slowly swaying towards the dactylozooid with the captured larva, and gradually opening its mouth on approaching the prey (Figure 3c; Video S1). The gastrozooid used the tentacles at its mouth region to capture the immobilized nauplius from the dactylozooid. The mouth of the gastrozooid expanded to its maximum state, while the nauplius was being slowly ingested, but closed immediately once the nauplius entered its gastrovascular cavity (Video S1).

When exposed to cyprids, the dactylozooids and gastrozooids were irresponsive, as both polyps remained in their respective pores throughout the settlement behaviors (Figure 3d, e). To further study the differences in responses, we simultaneously introduced both nauplii (stage II and VI) and different age groups of cyprids (1‐, 2‐ and 3‐day‐old) to the fire corals. In these trials, the stimulated polyps remain static without swaying towards the cyprids, while their response towards the nauplii remained as described above (Figure 3f, Video S2). Some gastrozooids and dactolyzooids even retracted back into their respective pores when approached by exploring cyprids (Video S2). Even if cyprids directly contacted the capitate tentacles of dactylozooids or gastrozooids (Video S2), this did not result in the incapacitation of the cyprids.

### 3.3 Settlement behaviors of *Wanella milleporae* larvae

**FIGURE 4:**
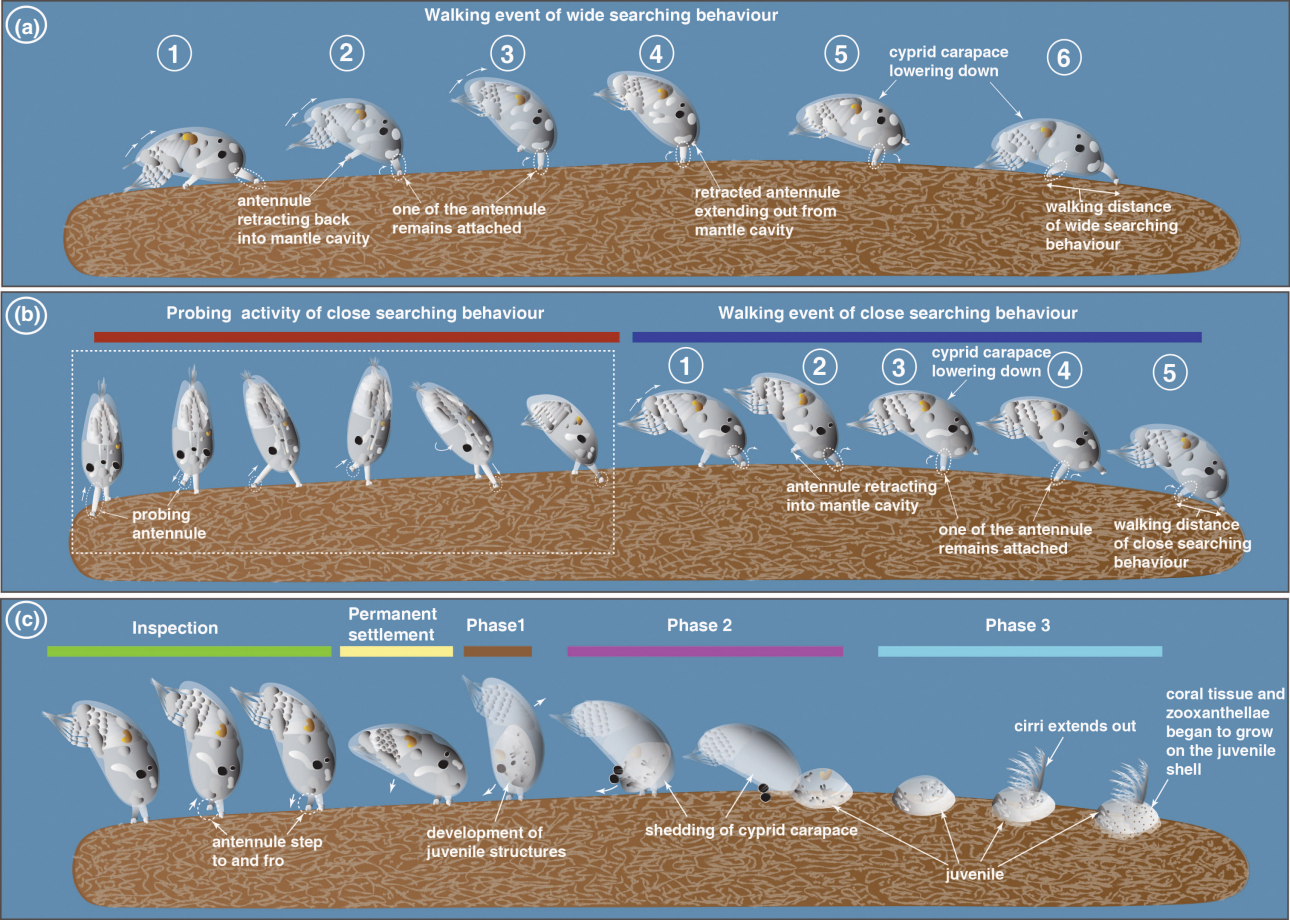
Schematic illustration of the cyprid settlement behaviors of *Wanella milleporae* on the fire coral, *Millepora tenera*. (a) The walking event in the wide searching behavior. Note the wide searching has larger step length than the close searching activity in (b). (b) Probing activity of antennules (red bar) and the walking event (blue bar) during the close searching behavior. (c) Final inspection (green bar), permanent settlement (yellow bar) and metamorphosis of cyprid (Phase 1 – brown bar, Phase 2 – purple bar and Phase 3 – light blue)

To elucidate settlement behaviors, we exposed newly developed cyprids (1‐day‐old) to the fire coral host. We found that the 1‐day‐old cyprids did not engage in surface exploration but swam around using their thoracopods without directly contacting the fire coral surface with their antennules. Exploration and settlement behaviors of cyprids were only observed from day 2 onwards. The cyprids began to swim towards the fire corals and initiated exploration behavior on the fire coral surfaces. At this point, the cyprids would lie almost flat with the setae of the thoracic limbs resting on the coral surface. Subsequently, they started to use their antennules to walk over the epidermal layer of the coral. This exploratory phase consists of three types of behaviors: wide searching, close searching and final inspection (Figure 4; Video S3–S7). The exploratory phase terminated when the cyprid attached permanently, indicating the initiation of metamorphosis into a juvenile barnacle (Figure 4, Video S8).

#### 3.3.1 Wide searching

This behavior featured prominently in 2‐ and 3‐day‐old cyprids and became less frequent as the cyprids aged. The *Wanella* cyprids walked by alternately extending and retracting the antennules, with one antennule temporarily attached to the coral surface. The antennules move like an inverted pendulum swing, causing the cyprid body to be slightly elevated (Figure 4a; Video S3). The cyprid then began to take a new walking step into its determined direction by extending the retracted antennule until it reached the maximum extension point to attach to a new spot on the fire coral surface. After completing a walking step, the cyprid body returned to lie almost flat on the fire coral with the setae of thoracic limbs touching the fire coral surface.

In wide search, slight directional changes occurred during the walking behavior when the cyprid encountered obstacles and uneven surface areas on the fire coral. The duration of the walking event varied between individuals. It was observed that the walking behavior could achieve up to an average distance of 1720 μm (~1315 to 1969 μm, *n* = 5) in 10 s. Each antennular step in the walking event ranged from 264 to 382 μm. The walking behavior ceased when an unfavorable condition was detected, leading to the detachment event. The cyprid would then swim off to locate and explore another potential surface area on the fire coral.

#### 3.3.2 Close searching

When a favorable site was detected, the cyprid initiated close searching behavior in search of a potential settlement site. The walking pattern in the close searching behavior is rather similar to the wide searching behavior with few evident alterations such as the probing behavior, rate of walking and a reduction in step length. The cyprid engaged in a more refined searching behavior, where the antennules were actively probing the surroundings on the fire coral surface before walking to the next potential attachment spot (Figure 4b, Video S4). The cyprid spent more time probing the fire coral surface with their antennules in close search, causing a relatively slower walking than in a wide search. The cyprids only walked an average distance of 520 μm (~176 to 839 μm, *n* = 10) in 10 s (average speed 52 μm s^−1^), almost three times slower than in wide search. The average distance of antennular steps in close search was also significantly different from the wide search (Welch unpaired *t*‐test, *p* < .001; Figure 7a). The walking behavior during the close search is frequently coupled with sidewise and reverse directional changes, called tight direction changes by Lagersson and Høeg (2002). These directional changes occurred when the cyprid detected an unfavorable attachment area (Video S5, S6).

#### 3.3.3 Inspection

Inspection behavior occurred when the surface explored by cyprid during close searching remained favorable (Figure 4c; Video S7). The walking ceased, and both antennules remained attached to a specific spot on the fire coral and moved in a jerking manner (Figure 4c; Video S7). The cyprid used the attachment disc to inspect the potential permanent settlement spot by stepping to and fro (i.e., forwards and backwards) on the fire coral. Attachment and detachment of the antennules frequently occurred at this point. Simultaneous with the jerking antennular movement, the cyprid body was pulled down by the antennules until the anterior end of the carapace touched the potential settlement area (Figure 4c; Video S7).

#### 3.3.4 Larval permanent settlement

Termination of the probing behavior of the antennules signals the start of the final and irreversible act of settlement (Figure 4c; Video S8). The cyprid body is oriented at an angle of about 30 to 45° to the coral surface, with the anteroventral region of the carapace resting on the coral surface. Momentary contractions of muscles associated with the thorax were evident during this phase, causing the involuntary movement of the cyprid carapace (Figure 4; Video S8). Although we did not observe the actual secretion process, the cyprid was attached irreversibly on the fire coral surface by secretion from the cement glands in the attachment organs (Figure 4c). The permanent attachment by cement leads to the onset of metamorphosis into the juvenile stage.

### 3.4 Metamorphosis in *Wanella milleporae* cyprids on the fire coral host.

**FIGURE 5:**
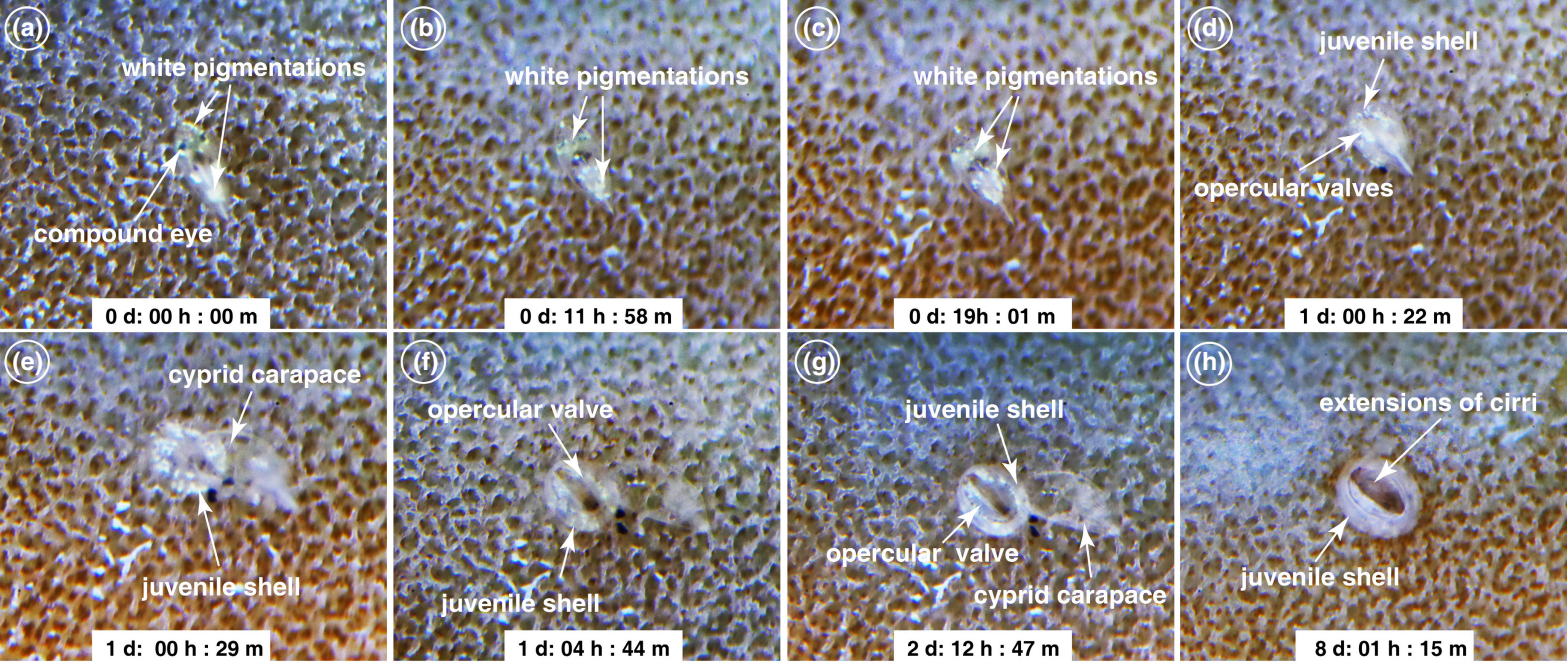
Metamorphosis of *Wanella milleporae* after settling on the fire coral. (a) Epidermal layer began to separate from the cuticle of cyprid. (b) Dissociation of the white pigmentation in the cyprid body. (c) The dissociated pigmentations converged in the anterior and posterior regions of the cyprid body. (d) Juvenile shell structure and opercular valves are visible beneath the cyprid body. (e) Juvenile cirri began to beat at this point to assist in shedding the cyprid carapace. (f) After carapace shedding, the basis of the juvenile appears to be attached to the fire coral. (g) The cuticle of juvenile shell structure becomes thick and the cirri remain inside the juvenile body. (h) The cirri are actively extending out after day 8

The metamorphosis resulted in an extreme morphological transition from a mobile larval stage (cyprid) into a sessile juvenile barnacle. The juvenile body developed beneath the cyprid cuticle still enclosing the metamorphosing individual (Figures 4c, 5; Videos S9–S11). The entire process took about 8 days (from cyprid permanent settlement until the extension of juvenile cirri) but varied among individuals. Based on our observations, the metamorphosis in *W. milleporae* can be described in three phases:

#### 3.4.1 Phase 1, pre‐transitional juvenile stage

After permanent settlement, minor twitching occurred sporadically within the cyprid body (Video S9), indicating the onset of the early metamorphic event. The juvenile epidermal layer slowly separated from the cuticle of the cyprid (Figure 5a, b; Video S9) in the first 12 h after settlement. Simultaneously, white pigmentations around the compound eyes and the thoraco‐abdomen dissociated and converged, respectively, to the anterior and posterior regions of the body (Figure 5b; Video S9). These white pigmentations and other cyprid cells became differentiated to form the juvenile body (Figure 5c; Video S9). The cyprid compound eyes migrated ventrally in the mantle cavity. About 24 h later, the juvenile shell structure and opercular valves were visible beneath the cyprid carapace (Figure 5d), and the body began twitching in the anterior region of the cyprid carapace.

#### 3.4.2 Phase 2, transitional stage

Although still enclosed in the cyprid carapace, the cirri of the juvenile started beating to assist the shedding of the old cyprid thoracopod cuticle. The shedding of the cyprid carapace was initiated when the body gradually rotated to a vertical stance (Figure 5c, e; Video S10). This rotation and continuous twitching ultimately cracked the cuticle in the anterior part of the cyprid carapace, resulting in the cyprid compound eyes being expelled, followed by the slipping out of the juvenile onto the attachment point (Figures 4c, 5f; Video S10). Failure to expel the compound eyes may affect the attachment of the juvenile onto the fire coral. From the video analysis, the basis of the newly developed juvenile appears to be inwardly folded before slipping out from the cyprid carapace. During the shedding of the cyprid carapace, the inwardly folded basis extended out from the cyprid and slowly spread out on the fire coral surface (Figures 4c, 5f; Video S10). This action served to increase contact surface area for attachment. When the cirri were beating, it was evident that the juvenile cuticle structure was very soft and moveable.

#### 3.4.3 Phase 3, post‐transitional stage

The mineralization of the shell structure occurred as the juvenile aged. The juvenile cirral remains beating for some time in the mantle cavity enclosed beneath the opercular valves (Figures 4c, 5g, Video S11). The extension of cirri only occurred at eight days after settlement (Figures 4c, 5h, Video S11).

### 3.5 Responses of fire corals towards metamorphosing *W. milleporae*

We observed two distinct host responses to the metamorphosing individuals of *W. milleporae*: non‐callus ring formation (Type 1) and callus ring formation (Type 2). Both responses involved cytotoxic incompatibility reaction of the fire coral towards the metamorphosing cyprid, but it differed significantly in the mortality rate of barnacles with 100% in Type 1 and 50% in Type 2 (Figure 7b).

**FIGURE 6:**
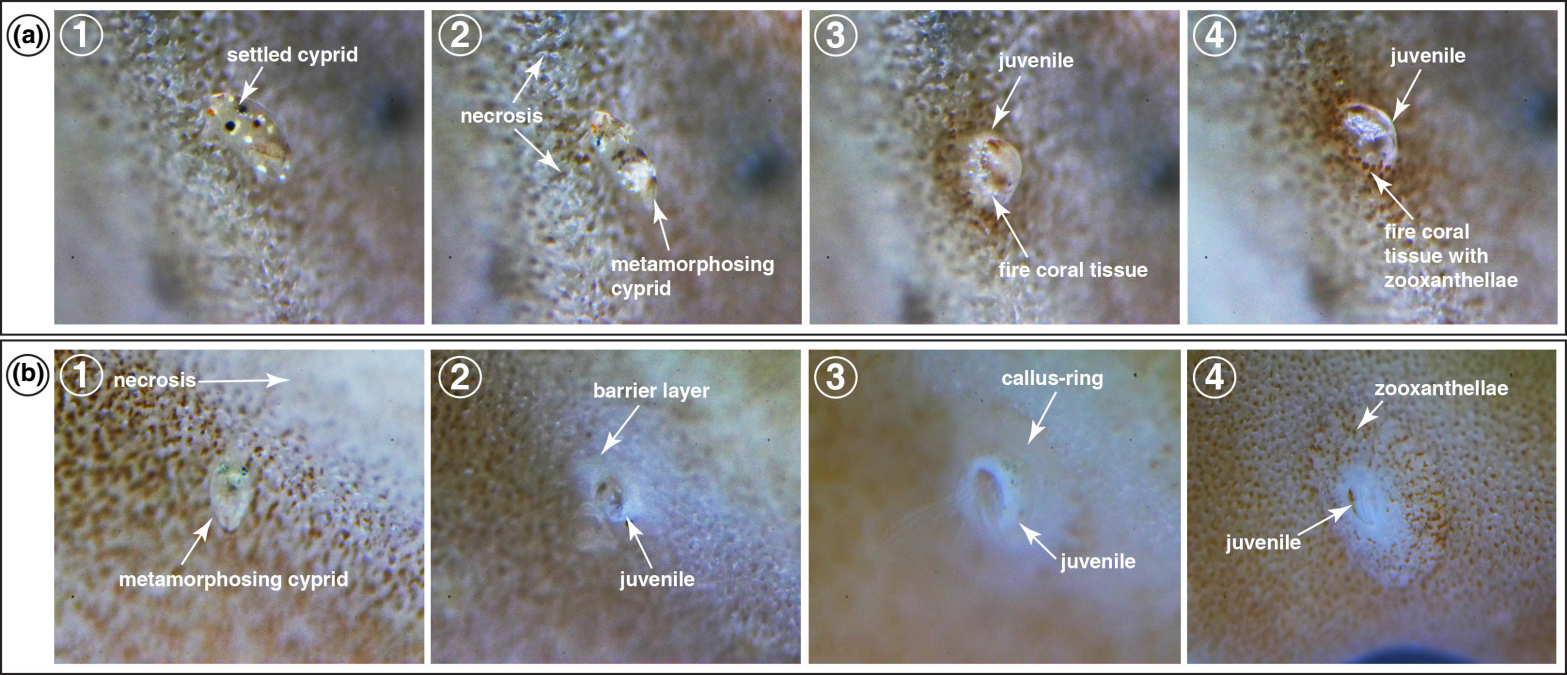
Xenogeneic reactions of fire coral in response to the metamorphosing cyprid of *Wanella milleporae*. (a) Type 1, non‐callus ring formation. (a1) Necrosis of the calcareous skeleton of fire coral around the metamorphosing cyprid. (a2) The necrosis event caused the disappearance of the zooxathellae, which leads to a change of color in the fire coral skeleton. (a3) Fire coral tissue began to grow and cover the juvenile shell structure. (a4) The fire coral tissue slowly regained zooxanthellae. (b) Type 2, callus ring formation. (b1) Necrosis process began with the surrounding of the metamorphosing cyprid and slowly reached the contact area between the fire coral and the cyprid. (b2) A barrier layer was formed in the contact point between the fire coral and the newly metamorphosised juvenile. (b3) Callus ring structure was formed around the juvenile shell structure. (b4) The callus ring structure covered the shell structure and regained the zooxanthellae

The histocompatibility reaction of fire coral was first initiated with the necrosis of the calcareous skeleton of fire coral around the metamorphosing cyprid. In Type 1, the necrosis only occurred in the surroundings of the metamorphosing cyprid (Figure 6a1). The necrosis event caused the discoloration of fire coral, indicating the loss or disappearance of the zooxanthellae (Figure 6a2).

After the cyprid successfully metamorphosed into a juvenile, a layer of fire coral tissue began to form on the juvenile shell structure (Figure 6a3). This layer of fire coral tissue slowly regained zooxanthellae and integrated with the juvenile shell structure (Figure 6a4). In some cases, the juvenile barnacle was entirely overgrown and covered by fire coral tissue, preventing the extension of feeding cirri, which inevitably led to the mortality of the barnacle.

In Type 2 response, the necrosis was progressive, and it started in the surroundings of the permanently settled cyprid and eventually reached the contact area between fire coral and barnacle (Figure 6b1). A barrier layer was formed on the necrotic area between the fire coral and the basis of the juvenile (Figure 6b2). This contact barrier layer slowly spread out to the contact point between fire coral and juvenile, forming a white circular‐shaped area. Once the spreading of the contact barrier layer ceased, the regeneration event of the fire coral was initiated, which ultimately led to the formation of a callus ring surrounding the entire shell armament of the juvenile barnacle (Figure 6b3). As the juvenile aged, the callus ring gradually regained zooxanthellae, reverting to its original state of fire coral (Figure 6b4), leaving the barnacle free to perform its cirral feeding. Such mechanisms were also observed in early settled individuals and juveniles in the field (Figure 1e, f).

### 3.6 Statistical analysis of larval age on the rate of settlement and walking behavior

**FIGURE 7:**
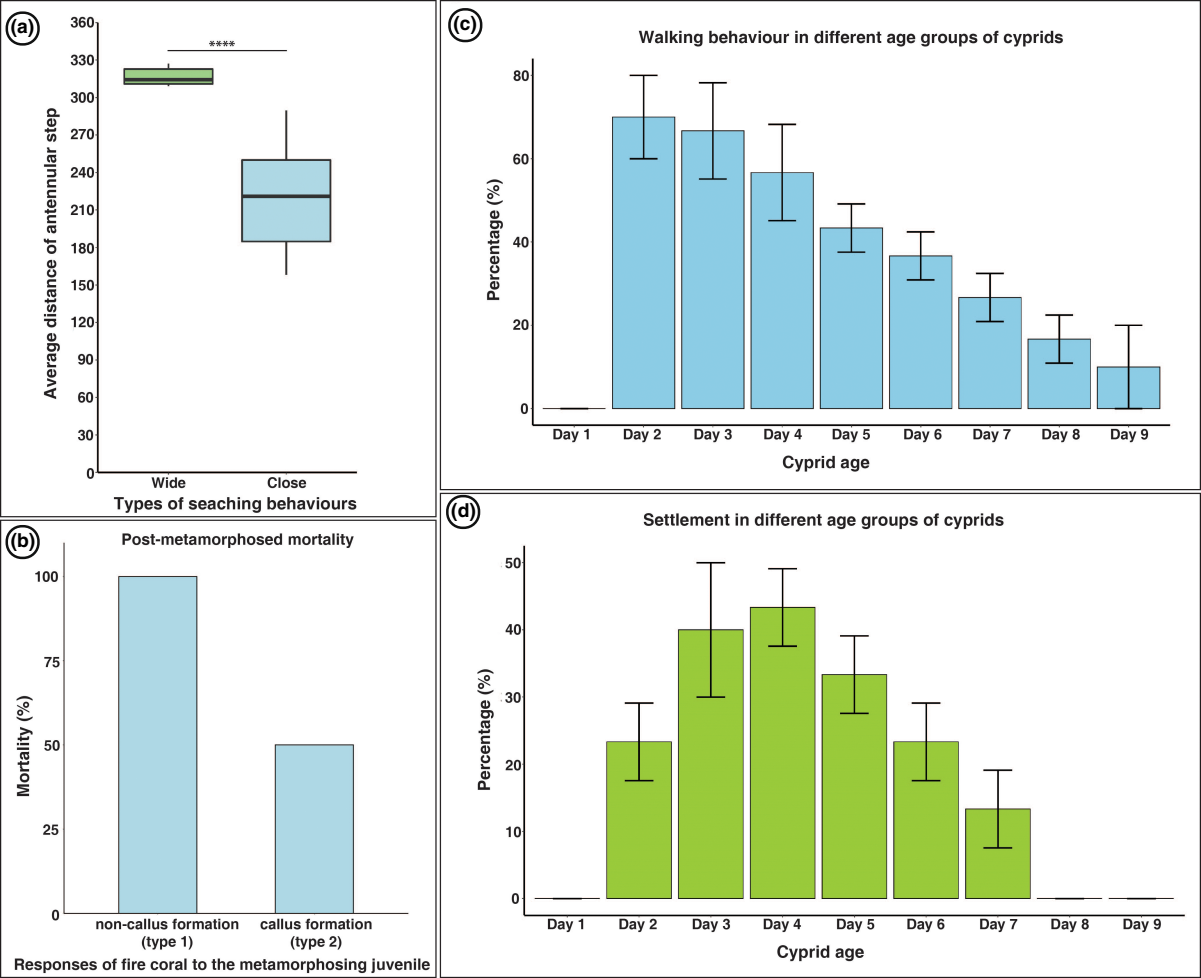
(a) Average distance of antennular step in the wide and close searching behaviors of *Wanella milleporae* (wide‐*n* = 5, close‐*n* = 10, Welch unpaired t‐test, *p* < .001). (b) Mortality rate of the juvenile in non‐callus ring formation (type 1) (*n* = 4) and callus ring formation (Type 2) (*n* = 4). (c) Rate of walking behavior in 1‐ to 9‐day‐old cyprids (*n* = 10, replicate = 3). (d) Settlement rate of cyprid in 1‐ to 9‐day‐old cyprids (*n* = 10, replicate = 3)

Different age groups of cyprids (1‐ to 9‐day‐old) have differed significantly in their walking activity (*F*_8,18_ = 28.18, *p* < 1.18e‐08, Figure 7c). Pairwise comparisons with Tukey HSD statistical test indicated that the walking activity in 2‐day‐old cyprid was significantly higher than the other age groups. Moreover, walking activity decreased significantly following this maximum. Similarly, cyprids from different age groups differed in the settlement rate (*F*_8,18_ = 30.22, *p* < 6.68e‐09, Figure 7d). The ad hoc Tukey HSD test showed that settlement first increased significantly with age, reaching a maximum in 4‐day‐old cyprids, but it decreased significantly after day 4 until there was no settlement, as in 8‐ and 9‐day‐old cyprids.

## 4 DISCUSSION

Symbiotic interactions of associated invertebrates with their specific hosts are an essential issue for promoting biodiversity in an ecosystem. In the marine environment, such symbiotic relationships are frequently initiated by the larvae of associated invertebrates that are biologically and structurally different from the ensuing adults. The task facing such larvae becomes more challenging when the host possesses defense mechanisms that can put them at risk of settling on the future host, such as the case in corals, sponges and fire corals. It remains largely unknown for the details of defense mechanisms, mainly due to the difficulty in culturing the larvae of associate and host together under laboratory conditions and successfully allowing the larvae to settle. In this study, we cultured the larvae of fire coral‐associated barnacles for the first time and observed successful settlement and metamorphosis of barnacle larvae on the host.

### 4.1 Planktonic larval phase of *Wanella milleporae*

Prior to settlement, the barnacle larvae need to disperse in the plankton through the free‐swimming naupliar stages and then metamorphose into cyprids, which are responsible for exploring the settlement substratum. These life cycle tasks of the larvae are common for all barnacles, irrespective of their biology and habitat (Anderson, 1994), but it becomes especially challenging in symbiotic species because they must locate their specific host to assure a successful relation. Chemical cues are important for planktonic larvae of the symbionts to detect and locate their hosts for settlement purposes (Gerlach & Atema, 2012; Lecchini & Nakamura, 2013; Pawlik, 1992; Zimmer & Butman, 2000). The sensory apparatus used in cyprid settlement is mainly located on the antennules, and it is therefore noteworthy that the armament of antennular sensory setae in cyprids of *W. milliporae* closely resemble those found in other barnacles (Bielecki et al., 2009; Dreyer et al., 2020). Therefore, we conclude that the ability to locate the host must rely on chemical specificity in the otherwise structurally similar sensory organs and by how the central nervous system processes these stimuli. We found that *W. milleporae* cyprids only landed on the surface of fire corals. When exposed to stony (scleractinian) corals, the cyprids never landed on their surfaces but remained swimming in the water column, i.e., they did not engage in a trial‐and‐error exploration of these non‐host species. This differs from the behavior seen in barnacles with a more generalized substratum preference, such as those from the rocky intertidal habitats. Hence, the cyprids of *W. milleporae* may be capable of using waterborne chemical stimuli to recognize their specific host at a distance and only initiate their exploratory behaviors when the actual host is present. Sponge inhabiting barnacles including *Balanus trigonus* and Acastinid barnacles, for example, can settle on sponges with the presence of chemical cues, including the metabolites (Haber et al., 2013; Yu et al., 2020). However, the free‐living *Amphibalanus amphitrite* cannot settle in the presence of the sponge metabolites (Haber et al., 2013). Similarly, the giant barnacle *Megabalanus*, octocorals and whip corals have inhibition cues to deter the non‐symbiont barnacle larvae from settling on their surfaces (Eswaran & Klandeparker, 2014; Rittschof et al., 1985; Standing et al., 1984). Sensing of hosts by waterborne chemical cues has also been argued for parasitic barnacles (Pasternak et al., 2004, 2005).

The next challenge is when the cyprids land on the fire coral surface as they have to overcome the defense mechanisms of fire corals or risk being predated. Fire corals are known to use their venom as anti‐predatory adaptations and to incapacitate the prey. The fact that the settlement of *W. milleporae* occurs in the larval stage has posed a potential danger of their larvae being incapacitated as prey by stinging polyps of the fire coral. Therefore, a mechanism to avoid contact with the stinging polyps would be essential for symbiotic barnacles. Intriguingly, we found that the polyps responded contrarily when exposed to nauplii and cyprids of *W. milleporae*. The presence of nauplii in the water column triggered the extension of the polyps and food capture behavior. In contrast, the fire coral polyps were irresponsive towards the cyprids and remained static or in their pores. Therefore, we suggest that cyprids of *W. milleporae* must have chemical mechanisms to suppress the activity of fire coral polyps, which allow them to move and walk freely without the risk of being immobilized and preyed upon. Moreover, there could be specialization in the general glandular apparatus of the cyprid (Chan et al., 2014; Høeg et al., 2004), which can result from a high degree of adaptive evolution into symbiosis with fire corals.

### 4.2 Attachment organs of *Wanella* cyprids

Barnacle cyprids are a specialized larval form of marine invertebrates, possessing a highly modified pair of antennules with a segmentation, musculature and terminal attachment device, that allow the cyprids to explore and walk on the substratum surfaces while searching for a suitable site to attach permanently (Aldred et al., 2013; Aldred & Clare, 2009; Lagersson & Høeg, 2002; Nott & Foster, 1969). Recent studies have suggested that the shape of the attachment disc on the third segment evolves adaptively in response to the characteristics of the settlement substratum, which can be truly observed in the coral and sponge barnacles (Brickner & Høeg, 2010; Yu et al., 2020). In coral‐associated barnacles, the cyprids possess spear‐shaped attachment discs that serve to penetrate the coral and ultimately allow the barnacle to become embedded in the host tissue (Brickner & Høeg, 2010; Liu et al., 2016). In contrast, we found that cyprids in the fire coral‐associated *W. milleporae* possess a relatively non‐specialized, bell‐shaped attachment disc, similar to those found in the barnacles inhabiting the rocky intertidal (Al‐Yahya et al., 2016; Bielecki et al., 2009; Chan et al., 2017; Nott & Foster, 1969). This similarity in the morphology of the attachment disc is probably due to the characteristic of the settlement surfaces. The ectodermal layer of fire coral appears to be a hard surface without a thick covering soft tissue. Hence, the barnacle, *W. milleporae*, does not need a specialized spear‐shaped attachment organ to penetrate the fire coral tissue.

### 4.3 Exploratory movements of cyprids on fire corals

In all barnacles, the cyprid is a non‐feeding larva specialized for settlement, and its survival relies on the amount of nutrients accumulated during the preceding naupliar phase or, if these are also non‐feeding, handed over from the egg (Hentschel & Emlet, 2000, Thiyagarajan et al., 2002). This obviously entails that a finite amount of energy is available for accomplishing the entire sequence of the host location, settlement and metamorphosis into a feeding juvenile. (Høeg et al., 2012; Maruzzo et al., 2012). In intertidal species, the “desperate larva” hypothesis (DLH) proposed that the older larvae can settle on any suboptimal settlement site rather than depleting the energy reserve and die without metamorphosis (Knight‐Jones, 1951, 1953; Toonen & Pawlik, 2001; Wilson, 1953). Our study is at odds with this hypothesis since the older cyprids of *W. milleporae* tend to lose their settlement competency and end their lifespans without metamorphosis. Rather, our results closely resemble the settlement pattern in the parasitic barnacle (Rhizocephala) studied by Høeg and Ritchie (1987). In both the parasitic barnacles and the fire coral symbiont *W. milleporae*, settlement activity in the cyprids declines with age. In such species, attachment on anything but the precise host animal would inevitably lead to death, explaining why the cyprids do not attempt settlement if this substratum is not found.

During wide searching, the cyprids walk at a relatively high speed and with long antennular step lengths to cover wide surface areas in search of a suitable settlement site. In close search, the speed and step length are vastly reduced, allowing more time to employ the antennular chemo‐ and mechanosensory setae to test the substratum (Crisp, 1976; Lagersson et al., 2003). The cyprid may also shift back and forth between these behaviors, when searching for a potential settlement site (Aldred et al., 2018; Crisp, 1976; Lagersson & Høeg, 2002).

The surface exploration in *W. milleporae* cyprids conformed to the pattern described above for wide and close searching behaviors. The movement pathways of *W. milleporae* are similar to paths in the Lévy walks, composed of short steps with longer steps between them (Benhamou, 2007; Reynolds, 2018). The Lévy walks created an unusual fractal movement pattern which is believed to be beneficial for searching, as it reduces needless walking or moving on the visited path (Reynolds, 2018). This movement pattern has been reported in various organisms such as marine predators (Sims et al., 2008), bacteria (Korobkova et al., 2004), jackals (Atkinson et al., 2002), dinoflagellates (Bartumeus et al., 2003), albatrosses (Humphries et al., 2013; Reynolds et al., 2015), honeybees (Reynolds et al., 2007), gray seals (Austin et al., 2004) and spider monkeys (Ramos‐Fernández et al., 2004). Therefore, the fractal properties of Lévy walks could benefit *W. milleporae* by allowing the cyprid to quickly access potential substratum sites for inspection before committing to settlement. However, the association of the movement path of *W. milleporae* with Lévy walks still requires further confirmation in statistical analyses and simulation to confirm this hypothesis (Abe, 2020). From a broader perspective, quantified exploration patterns should be compared across a wide spectrum of barnacle species. This would test the extent to which the morphologically very similar cyprids are behaviorally adapted to the highly specific substrata of their adults (Chan et al., 2017).

Most hermaphroditic barnacles need to settle within the copulatory range of conspecifics (Anderson, 1994; Zardus & Hadfield, 2000). But unlike the observations in many intertidal species (Head et al., 2004; Kent et al., 2003; Knight‐Jones, 1955), *W. milleporae* cyprids did not prefer settling extremely close to conspecifics. One possible explanation is that *W. milleporae* need more space to grow into the adult forms as they would be eventually covered by the fire coral tissues and their base embedded in the fire coral skeleton. Very often, the occurrence of *Wanella* is in so high abundance on fire corals that there are sexual partners within the mating distances (Figure 1). This differs from intertidal forms, where it is well known that the adults can form dense aggregations that may affect the shape of the house of shell plates (Bertness et al., 1998; Silina & Ovsyannikova, 2000).

Many animals have specific immunological competence, which allows them to recognize foreign invasions and eliminate them by effector mechanisms as a protection manner. The xenogeneic histocompatibility reactions are one of the recognition mechanisms in the fire coral, which is usually triggered when competing for space and light with other reef builders (Frank & Rinkevich, 1994; Müller et al., 1983). The xenogeneic encounters in fire corals involve detecting foreign tissue contact, necrosis of the contact area by autolysis process and regeneration events (Müller et al., 1983). These events allow the fire coral to recognize the invading foreign materials and protect themselves by forming a contact barrier layer as a preventive measure or eliminating the invader by overgrowth (Müller et al., 1983). Fire corals react similarly towards the metamorphosing *Wanella milleporae* cyprids, but there is variation in the subsequent regeneration events that significantly affect the post‐settlement mortality of the metamorphosed barnacles. Regeneration events of fire corals appear not to be an aggressive repelling response, but act as an integrative response. The rapid growth of fire coral tissue and formation of a callus ring structure on the juvenile barnacle suggest that the fire coral recognizes the barnacle, *W. milleporae,* as a harmless or positive symbiont, despite the occurrence of competition for space. Alternatively, *W. milleporae* may be able to control the coral growth after the callus ring is created. Therefore, the formation of the callus ring is a key process for the survival of the symbiotic barnacle (Figure 1e, f). Under the laboratory conditions, the mortality in the Type 2 response achieved 50%, which may be explained by some low‐fitness or dying juveniles cultured that still became overgrown by fire coral. We could not ascertain the percentage of mortality of recruits in Type 2 response in the field. From field observations, almost all *Wanella* on the fire coral hosts has the remnants of callus ring around its shell, suggesting the formation of a callus ring is essential for the subsequent survival of barnacles (Figure 1). It appears that the region of callus ring will subsequently grow upwards and the barnacles follow the same direction of longitudinal growth with the corals (Hiro, 1937), creating a shell cavity that houses the barnacle somatic body and is embedded inside the remnants of the callus ring (Figure 1e). The mortality of recruits in Type 2 response may be lower than that recorded at the laboratory in the present study.

### 4.4 Evolution of epibiotic movements and attachment organs

**FIGURE 8:**
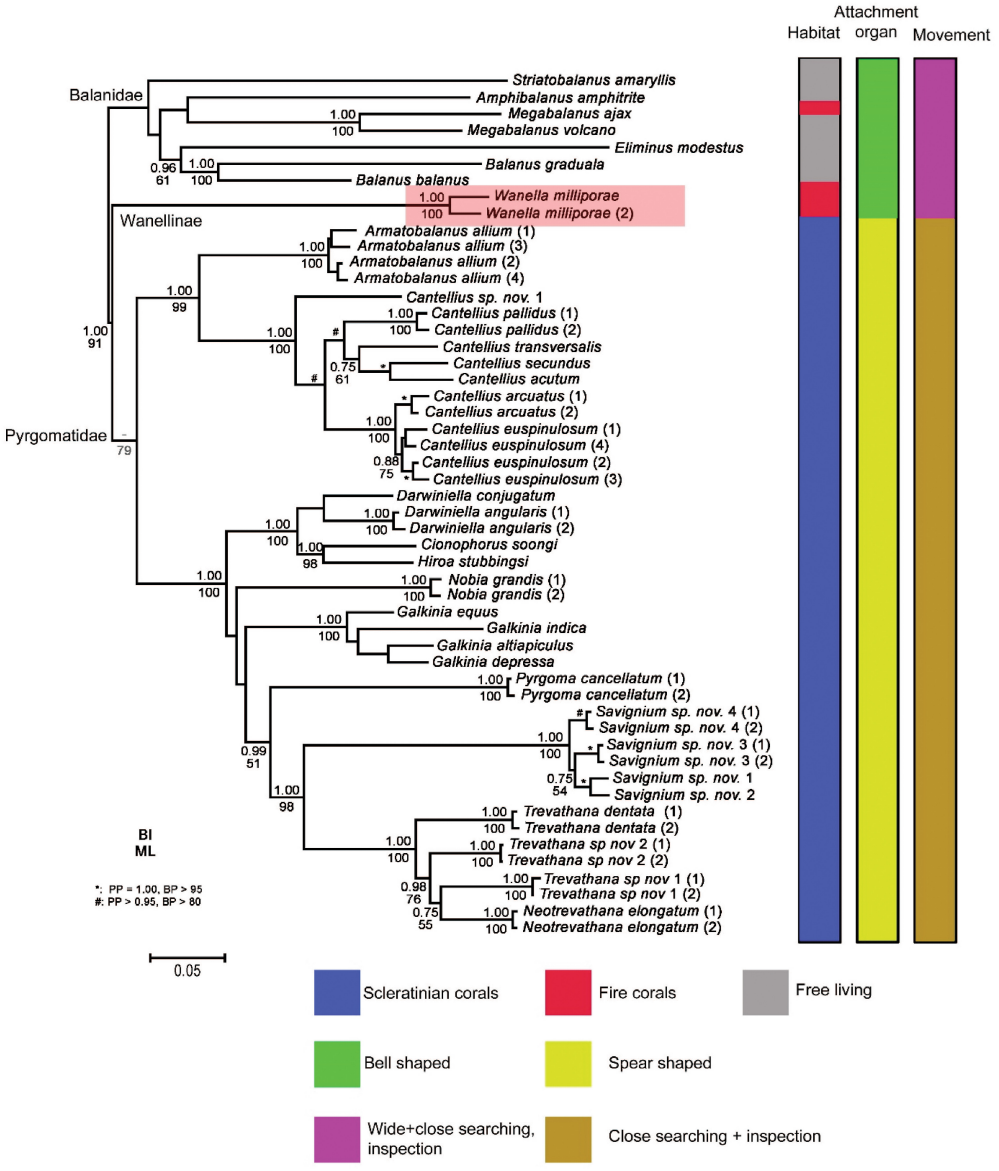
Phylogenetic tree based on multiple DNA markers adapted from Tsang et al. (2014) shows the phylogeny of coral, fire‐coral associated and free‐living barnacles. The study organisms *Wanella milleporae* are highlighted in red in the tree. Note the variation in habitats, shape of attachment organ and movement pattern of barnacles in the column pattern on the right side of the tree. The movement behaviour of Pyrgomatid barnacle cyprids is predicted based on the observations in Darwiniella angularis cyprids in Liu et al. (2016)

Several groups of barnacles are symbiotic with corals, fire corals and sponges in the coral reef ecosystem. From multiple DNA‐marker based molecular phylogenetic studies on barnacle phylogeny (Figure 8), coral‐associated barnacles and fire coral‐associated barnacles evolved independently into symbiosis from what it appears to have been intertidal forms (Tsang et al., 2014). It appears there is an adaptive radiation in the attachment organs in response to the settlement substratum. Within the large clade of acorn barnacles (Balanomorpha), a spear‐shaped attachment organ (third antennular segment) is mainly found in the coral‐associated barnacles of the monophyletic Pyrgomatidae and in the genus *Berndtia* in the order Acrothoracica, which are exclusively symbiotic in scleractinian corals (Brickner & Høeg, 2010; Chan et al., 2021; Dreyer et al., 2022; Liu et al., 2016; Yu et al., 2020; Figure 8). This spear‐shaped attachment organ serves to penetrate into the coral tissue, but it does not affect the surface exploration behavior, which resembles those found in most other barnacles. *Wanella milleporae* cyprids settle on fire corals with a hard skeleton, and thus, the cyprids possess a bell‐shaped attachment organ similar to those rocky intertidal species. Movement patterns in both types of cyprids also comprised the same wide search, close search and inspection behaviors (Aldred et al., 2018; Lagersson & Høeg, 2002). Yet, we predict that a future close analysis may show that the time spent for these three phases may differ between symbiotic and intertidal barnacle species. Presumably, symbionts would chemically recognize that they are on the suitable surface and thus spend less time in close search to find the final site for cementation. An extreme case of chemical‐based host recognition has been documented in parasitic forms (Rhizocephala), where the entire sequence of host location, surface exploration and metamorphosis can be completed within a few minutes (Høeg, 1985). Further mapping of settlement behavioral patterns onto the phylogenetic tree may thus well show adaptive evolution driven by host, as already demonstrated for the morphology of the antennular attachment organ. Examples of host‐driven adaptive radiations were found in a number of symbiotic relationships including butterflies and plants, and Nudibranchs and sponges. In the butterfly–plant interactions, plant biochemistry is the major factor to govern the evolution and diversification of the butterflies. The degree of chemoreceptive responses and the ability for physiological tolerance to different plant secondary substance in butterfly larvae can result in adaptive radiation events (Ehrlich & Raven, 1964). Plants often use metabolites including alkanoid compunds to deter insect grazing. However, herbivores can evolve resistance to those compounds and can sequester metabolites in body tissues to avoid them from being further predated by predators (Ehrlich & Raven, 1964). From the molecular phylogenetic analysis, the *Adelpha* and the *Parnassius* butterflies have monophyletic molecular clades that contain species that exclusively feed on alkanoid containing plants, resulting from adaptive radiation events (Ebel et al., 2015; Rebourg et al., 2006). Chromodorid nudibranchs exclusively feed on sponges that contain high levels of secondary metabolites. Rogers and Paul (1991) found that *Glossodoris* nudibranchs can excrete or alter some of the secondary metabolites from the sponge preys. Molecular phylogenetic studies showed that Chromodorididae is a monophyletic group suggesting the mechanisms for tolerance of secondary metabolites from sponges are a result of adaptive radiation. In the molecular barnacle phylogenetic tree (see Chan et al., 2021; Tsang et al., 2014), *Wanella* is composed of a single genus monophyletic clade which appears to be a result of adaptive radiation, similar to the mechanisms observed in butterflies and nudibranchs.

Our study demonstrated that a positive interaction might have occurred between *W. milleporae* and their fire coral hosts, allowing the cyprids to explore unimpeded by nematocyst defenses and subsequently metamorphose and become surrounded by a callus ring structure formed by the fire coral host as an integrative process. We further advanced how this symbiosis is initiated by adopting a movement ecology framework to study how symbiotic larvae move, settle and start the symbiotic life with their fire coral hosts.

## AUTHOR CONTRIBUTIONS

**Fook‐Choy Yap:** Conceptualization (equal); data curation (equal); formal analysis (equal); investigation (equal); methodology (equal); software (equal); writing – original draft (equal); writing – review and editing (equal). **Jens T. Hoeg:** Conceptualization (equal); formal analysis (equal); investigation (equal); writing – original draft (equal); writing – review and editing (equal). **Benny K. K. Chan:** Conceptualization (equal); data curation (equal); formal analysis (equal); investigation (equal); methodology (equal); writing – original draft (equal); writing – review and editing (equal).

## CONFLICT OF INTEREST

The authors declare no conflict of interest.

## Supporting information


Videos S1‐S11

## Data Availability

All supplementary videos (Video S1–S11) are available on the Figshare repository at https://figshare.com/s/c5a226d322f1f2e7dc22.
