# Supplementary material for: Living on fire: Deactivating fire coral polyps for larval settlement and symbiosis in the fire coral‐associated barnacle Wanella milleporae (Thoracicalcarea: Wanellinae)
Source: Ecol Evol. 2022 Jul 5;12(7):e9057. doi: 10.1002/ece3.9057 (PMC9254672; doi:10.1002/ece3.9057)
Supplement: Supplementary file 1 — Videos S1‐S11 [file ECE3-12-e9057-s001.docx]

Electronic supplementary materials

All videos can be directly viewed or downloaded from the Figshare repository at <https://figshare.com/s/c5a226d322f1f2e7dc22>

**Video** **S1** Behavioral responses of fire coral polyps against the barnacle nauplii of *Wanella milleporae*.

**Video** **S2** Behavioral responses of fire coral polyps against the barnacle cyprid of *Wanella milleporae*.

**Video** **S3** Exploratory behavior of *Wanella milleporae* cyprid on the fire coral, *Milleporae tenera* – Wide searching.

**Video** **S4** Exploratory behavior of *Wanella milleporae* cyprid on the fire coral, *Milleporae tenera* – Close searching.

**Video** **S5** Sidewise directional change during the close searching behavior of *Wanella milleporae* cyprid.

**Video** **S6** Reverse directional change during the close searching behavior of *Wanella milleporae* cyprid.

**Video** **S7** Inspection behavior of *Wanella milleporae* cyprid on the fire coral surface.

**Video** **S8** Permanent settlement of *Wanella milleporae* cyprid on the fire coral surface.

**Video** **S9** Phase 1 – Early metamorphosis of *Wanella milleporae* cyprid on the fire coral, *Millepora tenera*.

**Video** **S10** Phase 2 – Shedding of cyprid carapace of *Wanella milleporae* during cyprid metamorphosis

**Video** **S11** Phase 3 – Newly metamorphosed juvenile of *Wanella milleporae*.
